# Multicenter, phase III trials on the contraceptive efficacy, tolerability and safety of a new drospirenone‐only pill

**DOI:** 10.1111/aogs.13688

**Published:** 2019-08-06

**Authors:** Santiago Palacios, Enrico Colli, Pedro‐Antonio Regidor

**Affiliations:** ^1^ Instituto Palacios Salud y Medicina de la Mujer Madrid Spain; ^2^ Exeltis HealthCare Madrid Madrid Spain; ^3^ Exeltis Europe Ismaning Germany

**Keywords:** drospirenone, drospirenone‐only pill, estrogen‐free contraception, Pearl index, venous thromboembolic events

## Abstract

**Introduction:**

Approximately 100 million women currently use combined oral contraceptives. Combined oral contraceptives use is associated with increased risk of venous thromboembolic events and cardiovascular disease. Progestin‐only pills do not increase the risk of venous thromboembolic events, stroke and myocardial infarction but are associated with a poor cycle control. A novel estrogen‐free pill containing only drospirenone (DRSP) was developed to improve bleeding pattern, tolerability and acceptance without increasing venous thromboembolic events risks in contraception.

**Material and methods:**

Two prospective, multicenter Phase III studies in healthy women aged 18‐45 years were performed to demonstrate the efficacy and safety of a drospirenone‐only pill in a regimen of 24 days of 4 mg of drospirenone tablets followed by 4 days of placebo. A total of 1571 women (14 329 exposure cycles) were analyzed: 713 patients in the 13‐cycle study 1 with 7638 exposure cycles and 858 patients in the 9‐cycle study 2 with 6691 exposure cycles. The primary endpoint was the overall Pearl index, calculated for each study separately, and for both pooled. As main secondary efficacy endpoint, the “method failure Pearl index” including all pregnancies during “perfect medication cycles” was evaluated. EudraCT registration numbers: 2010‐021787‐15 & 2011‐002396‐42.

**Results:**

Calculations on pooled studies 1 and 2 with 1571 patients gave an overall Pearl index (based on 14 329 cycles) of 0.7258 (95% CI 0.3133 to 1.4301). No single case of deep vein thrombosis or pulmonary embolism and only one case of hyperkalemia were reported. Additional information such as laboratory parameters, body mass index, bodyweight, heart rate and blood pressure showed no statistically significant changes due to the treatment.

**Conclusions:**

This is the first report of a new drospirenone‐only oral contraceptive providing clinical efficacy similar to combined oral contraceptives, with a good safety profile, and favorable cycle control.

**Abbreviations**
AE: adverse event
CI: confidence interval
COC: combined oral contraceptive
DRSP: drospirenone
PI: Pearl index
POP: progestin‐only pills
VTE: venous thromboembolic events

**Key message**

A new estrogen‐free contraception containing 4 mg drospirenone in a 24/4 cycle regimen was shown to be a safe and effective option with a Pearl index of 0.72 improving cycle control in comparison with established estrogen‐free contraceptives.

## 1 INTRODUCTION

Various effects of progestogens, in addition to inhibition of ovulation, have been identified, such as protection against endometrial and ovarian cancer, relief of dysmenorrhea and endometriosis symptoms, and decreased menstrual flow.^1^ Progestogens have little impact on the coagulation system and their effects on blood flow and contractility of vessel walls are limited.^2^ Epidemiological and clinical studies do not show any significant risk for thromboembolic venous or arterial disease.^3^, ^4^, ^5^ Therefore, progestogen‐only contraceptives can be used in women for whom combined hormonal contraceptives are contraindicated (World Health Organization [WHO] Medical Eligibility Criteria [MEC] for contraceptive use, Category 4) or where the use of COC is not advised (WHO MEC, Category 3).^4^ The most frequent side effect of the continuous use of progestogens is irregular bleeding.^6^

The progestogen‐only pill (POP) containing 75 μg desogestrel daily is taken continuously without a 7‐day break. Desogestrel inhibits ovulation and is as effective as combined hormonal contraceptives. No major health risks are known, and it has a Pearl index (PI) like COCs. It also alleviates menstrual migraine, pain in patients with endometriosis, and hypermenorrhea and dysmenorrhea.^7^, ^8^, ^9^

Irregular bleeding together with a stringent daily timing and missed pill rules may affect contraceptive reliability. For these reasons, despite their safe and efficacious profile, POPs are still not widely used and there is a need for new estrogen‐free products. A new estrogen‐free contraceptive containing 4 mg drospirenone (DRSP) has been developed to address this need.

Drospirenone is a unique progestogen derived from spirolactone that closely matches the properties of progesterone. It has anti‐mineralocorticoid and antiandrogenic properties.^10^ A 4‐mg dose of DRSP was selected after completion of pharmacokinetic and pharmacodynamic studies.^11^ Multiple exposure to DRSP 4 mg demonstrated a lower systemic exposure of DRSP compared with 20 mg ethinylestradiol/3 mg DRSP. Additional testing with 4 mg DRSP demonstrated inhibition of ovulation similar to desogestrel 75 μg for two 28‐day cycles.^10^, ^12^ Although the dosage of the new formulation is higher than the dosage of other POP when compared to their dosage in COC, no safety concerns are expected, as ethinylestradiol is known to be a potent inhibitor of CYP3A4 and of SULT1A1, resulting in a significantly higher serum level of DRSP in combined formulations.^13^, ^14^

The aim of the presented studies was to assess the contraceptive efficacy of the DRSP‐only pill and to provide safety information. Tolerability regarding a bleeding pattern in comparison with the existing POP was a secondary aim of these trials.

## 2 MATERIAL AND METHODS

Two prospective, multicenter phase III studies were performed to demonstrate the efficacy and safety of a DRSP‐only contraceptive pill. Both studies took place in Europe. Study 1 included 41 centers located in Czech Republic, Germany, Hungary, Poland and Romania. Study 2 was a double‐blinded, randomized controlled trial including 88 centers in Austria, Czech Republic, Germany, Hungary, Poland, Romania, Slovakia and Spain. The protocol was designed and conducted according to existing legal regulations and in accordance with good clinical practice in the conduct of clinical trials and the Declaration of Helsinki. Data from both studies were used for analysis of the primary and secondary endpoints.

### 2.1 Study medication

The study medication was one tablet containing 4 mg non‐micronized DRSP per day, via oral route, with consecutive administration of 24 active tablets and four placebo tablets, and no tablet‐free interval between two consecutive cycles.

Desogestrel 0.075 mg (in a regimen of 28 active pills) was the comparator for safety in study 2. Randomization to DRSP or desogestrel in a 5:2 ratio was performed by a contract research organization (Scope International AG, Mannheim, Germany) using a validated system that automates the random assignment of treatment groups to randomization numbers. During the study, the subjects and all personnel involved in the conduct and interpretation of the trial, were blinded to the medication codes. Compliance was measured using an electronic diary.

### 2.2 Duration of studies

The duration of treatment intake in study 1 was 13 cycles of 28 days, with a follow‐up visit without treatment 10‐28 days after the last study medication. The duration of treatment intake in study 2 was 9 cycles of 28 days, with a follow‐up visit without treatment performed 7‐10 days after the last study medication.

### 2.3 Study populations

Inclusion criteria for these studies were: women of child‐bearing potential, at risk of pregnancy, agreeing to use only the study medication for contraception for the duration of the study treatment, aged 18‐45, with a systolic blood pressure <140 mmHg and a diastolic blood pressure <90 mmHg.

### 2.4 Primary efficacy endpoint

The primary efficacy criterion was the overall Pearl Index (PI), calculated as follows:

Overall PI=number of pregnancies×1300/number of medication cycles.

The overall PI included all pregnancies which occurred during the study. Pregnancies following premature termination of the study medication were excluded from calculations.

### 2.5 Secondary efficacy endpoints

The “method failure PI” included all pregnancies during “perfect medication cycles”; defined as sexually active cycles without additional contraception where the e‐diary documented regular pill intake during the scheduled active cycle period (days 1‐24), excluding cycles with ≥4 days forgotten tablets/missed diary entries, ≥2 consecutive days with forgotten tablets/missed diary entries in the active cycle period or protocol deviations affecting the cycle.

### 2.6 Safety

Adverse events (AEs) reported by the women or observed by the clinical investigator during the study were registered using the case report form (CRF), including duration, causality assessed by investigator, seriousness, severity, frequency, treatment, action taken and outcome. Deep vein thrombosis or pulmonary embolism and hyperkalemia were considered AEs of special interest and led to discontinuation.

### 2.7 Sample size

According to European Medicines Agency requirements, the overall PI may be evaluated from more than one study.^12^ For an assumed PI <1.0, the number of cycles needed to fulfill the precision requirement with 90% power is 12.337. Thus 6169 cycles should be collected in each of the two studies, requiring 685 evaluable subjects with a treatment duration of 13 cycles in study 1 and 9 cycles in study 2.

Study 1 was performed to have at least half the evaluable cycles needed for an assumed PI <1.0 with a power of 90%. Considering a possible drop‐out rate of 25%, 700 women were to be enrolled in the study to give ≥6169 evaluable cycles. The cycles were collected such that ≥400 women would have 13 cycles, as specified in European Medicines Agency Guidelines.^12^

In study 2, to test non‐inferiority of the bleeding pattern between the two treatment groups (assuming a 24% proportion of the control group, 9% non‐inferiority margin, one‐sided type I error 2.5, 80% power, and 2:1 treatment allocation rate) a sample size of 531 in the DRSP group and of 266 in the desogestrel group was required. To prove superiority under the same assumptions, a sample size of 443 in the DRSP group and of 222 in the desogestrel group was required. Considering a possible drop‐out rate of 20%, to attain a 5:2 ratio, 857 DRSP and 333 desogestrel‐treated women should be enrolled.

### 2.8 Statistical analyses

In each study, efficacy analyses were performed on the full‐analysis set (FAS), defined as all subjects who took at least one dose of the study medication and who were not pregnant at entry.

The primary and secondary efficacy endpoints were calculated for each study separately and for both studies pooled, as well as their 95% CI.

For safety analyses, all AEs were coded according to MEDDRA version 15.0. All AEs were summarized using default summary statistics calculated from the number and percentage of subjects with AEs according to primary MEDDRA system organ class (SOC) and preferred term (PT). Summary and incidence of AEs were to be presented for each subgroup as well.

For tolerability analyses in study 2, rates of overall or unscheduled bleeding/spotting were compared between treatment groups using chi‐square tests. Significance between treatment groups was documented with a *P* value of <0.001.

### 2.9 Ethical approval

An ethical approval was obtained for each of the investigational centers. The overall approval for the study 1 with the leading ethical committee was given on 01 July 2011 by the Ethikkommission des Landes Sachsen‐Anhalt, Geschäftsstelle, number 10/0607 EK. The overall approval for the study 2 with the leading ethical committee was given on 13 July 2012 by the Landesamt für Gesundheit und Soziales Berlin, Geschäftstelle der Ethik Kommission des Landes Berlin, number 11/0606 EK. The EudraCT Numbers are 2010‐021787‐15 & 2011‐002396‐42. The dates on which the records were first entered in the EudraCT database were between 5 November 2010 and 14 April 14. Since 22 March 2011, the EudraCT has been open and publicly available. The first subject was entered on 11 July 2011.

## 3 RESULTS

### 3.1 Baseline characteristics

**Figure 1:**
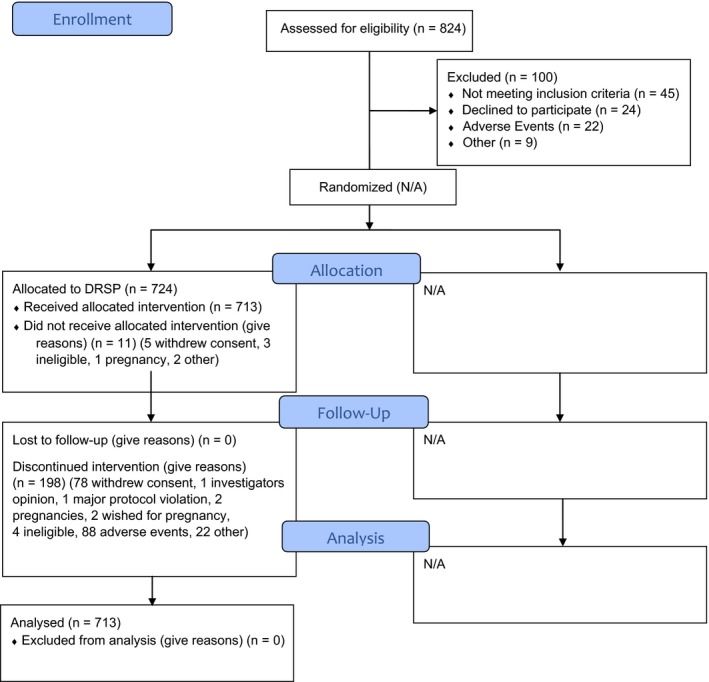
CONSORT 2010 Flow Diagram—Study 1. DRSP, drospirenone [Color figure can be viewed at http://www.wileyonlinelibrary.com]

**Figure 2:**
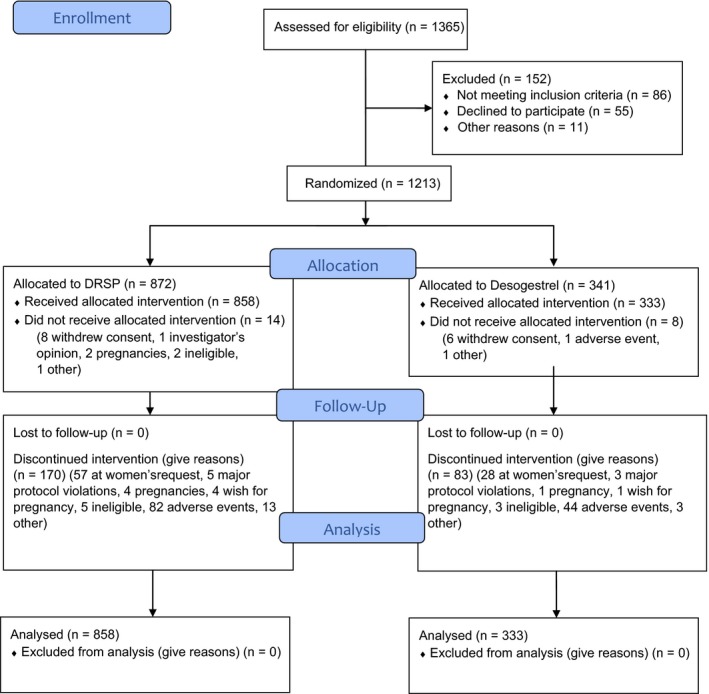
CONSORT 2010 Flow Diagram—Study 2. DRSP, drospirenone [Color figure can be viewed at http://www.wileyonlinelibrary.com]

A total of 1571 women (14 329 exposure cycles) were treated with 4 mg DRSP: 713 patients in the 13‐cycle study 1 with 7638 exposure cycles and 858 patients in the 9‐cycle study 2 with 6691 exposure cycles (see Figures 1 and 2). Demographic data are presented in Table 1.

**Table 1:** Baseline patients characteristics—Studies 1 and 2

	Statistics	Study 1	Study 2	
		DRSP 4 mg (n = 713)	DRSP 4 mg (n = 858)	Desogestrel 0.075 mg (n = 332)
Age (years)	Mean (SD)	28.7 (7.1)	28.9 (7.1)	28.9 (7.1)
Age group				
≤35 years	n (%)	569 (79.8%)	682 (79.5)	259 (78.0)
>35 years	n (%)	144 (20.2%)	176 (20.5)	73 (22.0)
Ethnicity				
Caucasian	n (%)	710 (99.6%)	856 (99.8)	331 (99.7)
BMI (kg/m^2^)	Mean (SD)	23.0 (3.8)	22.96 (3.537)	22.82 (3.905)
	Min/Max	16/38	16.6/41.0	15.9/38.0
BMI group				
<30 kg/m^2^	n (%)	672 (94.2%)	828 (96.5)	316 (95.2)
≥30 kg/m^2^	n (%)	41 (5.8%)	30 (3.5)	16 (4.8)
BP group				
SBP <130 and DBP <85 mmHg	n (%)	571 (80.1%)	727 (84.7)	290 (87.3)
SBP ≥130 and DBP ≥85 mmHg	n (%)	142 (19.9%)	131 (15.3)	42 (12.7)
Subject status				
Switcher	n (%)	391 (54.8%)		
Direct switcher	n (%)	—	628 (73.2)	259 (78.0)
Indirect Switcher	n (%)	—	39 (4.5)	14 (4.2)
Starter	n (%)	309 (43.3%)	191 (22.3)	59 (17.8)
Unknown	n (%)	13 (1.8%)	—	—
VTE risk factor				
Presence of at least one risk factor	n (%)	110 (15.4%)	142 (16.5)	59 (17.8)
Previous delivery				
Yes	n (%)	305 (42.8%)	395 (46.0)	150 (45.2)
Regular menstrual bleeding during the last 6 cycles				
Yes	n (%)	680 (95.4%)	786 (91.6)	305 (91.9)
Prior treatment with sex hormones and modulators of the genital system				
Yes	n (%)	455 (63.8%)	469 (54.7)	195 (58.7)

Abbreviations: BMI, body mass index: BP, blood pressure; DBP, diastolic blood pressure; DRSP, drospirenone; SBP, systolic blood pressure; VTE venous thromboembolic events.

### 3.2 Efficacy based on the primary and secondary endpoints in the whole population

During study 1 (713 patients with 7638 cycles) three patients became pregnant, with estimated conception dates in cycles 2, 3 and 13, leading to an overall PI of 0.5106 (95%CI 0.1053 to 1.4922). All three pregnancies were considered treatment failures. The overall PI after correction for additional contraception and sexual activity status (based on 7191 cycles) was 0.5423 (95%CI 0.1118 to 1.5850). The method failure PI (based on 6101 perfect medication cycles) was 0.6392 (95%CI 0.1318 to 1.8681). The cumulative 13‐cycle pregnancy ratio was 0.50% (95%CI 0 to 1.07%).

During study 2 (858 patients with 6691 DRSP cycles), five patients using 4 mg DRSP (0.6%) became pregnant with estimated conception dates in cycles 3, 5, 6, 8 and 9. The overall PI was 0.9715 (95%CI 0.3154 to 2.2671). All five pregnancies were considered treatment failures. The overall PI after correction for additional contraception and sexual activity status (based on 5977 cycles) was 1.0875 (95% CI 0.3531 to 2.5379). The method failure PI (based on 4641 perfect medication cycles) was 1.4006 (95%CI 0.4548 to 3.2684). The cumulative 9‐cycle pregnancy ratio was 0.70% (95%CI 0.09 to 1.31).

Calculations on pooled studies 1 and 2 (1571 patients with 14 329 cycles) gave an overall PI of 0.7258 (95%CI 0.3133 to 1.4301). All eight pregnancies were considered treatment failures. The overall PI after correction for additional contraception and sexual activity status (based on 13 168 cycles) was 0.7898 (95%CI 0.3410 to 1.5562). The method failure PI (based on 10 742 perfect medication cycles) was 0.9682 (95%CI 0.4180 to 1.9077). The cumulative 13‐cycle pregnancy ratio of DRSP users in both trials was 0.72% (95%CI 0.17 to 1.27). Results of separate and pooled analyses are presented in Table 2.

**Table 2:** Efficacy results in study 1, study 2 and pooled analysis

	Statistics	Study 1	Study 2		Pooled analysis
		DRSP 4 mg (n = 713)	DRSP 4 mg (n = 858)	Desogestrel 0.075 mg (n = 332)	DRSP 4 mg Total (n = 1.571)
Overall Pearl index					
Total number of exposure cycles	n	7.638	6.691	2.487	14.329
Pregnancy	n (%)	3 (0.4%)	5 (0.6%)	1 (0.3%)	8 (0.5%)
Overall Pearl index	%	0.5106	0.9715	0.5227	0.7258
95%CI	Lower limit/upper limit	0.1053/1.4922	0.3154/2.2671	0.0132/2.9124	0.3133/14301
Overall Pearl index after correction for additional contraception and sexual activity status					
Total number of cycles with sexual activity and without additional contraception	n	7.191	5.977	2.224	13.168
Pregnancy	n (%)	3 (0.4%)	5 (0.6%)	1 (0.3%)	8 (0.5%)
Overall Pearl index after correction for additional contraception and sexual activity status	%	0.5423	1.0875	0.5845	0.7898
95%CI	Lower limit/upper limit	0.1118/1.5850	0.3531/2.5379	0.0148/3.2568	0.3410/1.5562
Method failure Pearl index					
Total number of perfect medication cycles	n	6.101	4.641	1.816	10.742
Pregnancy	n (%)	3 (0.4%)	5 (0.6%)	1 (0.3%)	8 (0.5%)
Method failure Pearl index	%	0.6392	1.4006	0.7159	0.9682
95%CI	Lower limit/upper limit	0.1318/1.8681	0.4548/3.2684	0.0181/3.9885	0.4180/1.9077
Overall pregnancy ratio					
	%	0.50%	0.70%	0.34%	0.72%
95%CI	Lower limit/upper limit	0.00/1.07%	0.09/1.31%	0.00/1.01%	0.17/1.27%

Abbreviation: DRSP, drospirenone.

The baseline characteristics of the eight pregnant women were identical to the non‐pregnant women. No ectopic pregnancy was recorded.

### 3.3 Efficacy based on the primary and secondary endpoints in women aged ≤35 years

During study 1, all pregnancies occurred in women aged *≤*35 years. In this subgroup (569 patients with 5915 cycles), the overall PI was 0.6593 (95%CI 0.1360 to 1.9269), the overall PI after correction for additional contraception and sexual activity status (based on 5530 cycles) was 0.7052 (95%CI 0.1454 to 2.0610) and the method failure PI (based on 4646 perfect medication cycles) was 0.8394 (95%CI 0.1731 to 2.4532). The cumulative 13‐cycle pregnancy ratio was 0.64% (95%CI 1‐1.37%).

During study 2, all pregnancies occurred in women aged *≤*35 years. In this subgroup (682 patients with 5230 cycles), the overall PI was 1.2428 (95%CI 0.4035 to 2.9004). The overall PI after correction based on 4643 cycles was 1.4000 (95%CI 0.4546 to 3.2670) and the method failure PI (based on 3542 cycles) was 1.8351 (95%CI 0.5959 to 4.2826). The cumulative 9‐cycle pregnancy ratio was 0.90% (95%CI 0.11 to 1.68%).

In women aged ≤35 years, calculations on pooled studies 1 and 2 (1251 patients with 11 145 cycles) gave an overall PI of 0.9332 (95%CI 0.4029 to 1.8387). All eight pregnancies were considered treatment failures. The overall PI after correction for additional contraception and sexual activity status (based on 10 173 cycles) was 1.0223 (95%CI 0.4414 to 2.0144). The method failure PI (based on 8188 perfect cycles) was 1.2702 (95%CI 0.5484 to 2.5027). The cumulative 13‐cycle pregnancy ratio of DRSP users in both trials was 0.93 (95%CI 0.21 to 1.64).

### 3.4 Bleeding profile

In both studies, and in all treatment groups, there was a decrease over time in the overall number of patients with bleeding or spotting and in the number of unscheduled bleeding or spotting. The highest rates were observed during the first reference period, Cycle 2 to Cycle 4, in all studies and treatment groups. There was a significantly lower rate in the DRSP 4 mg group as compared with the desogestrel group in study 2 (79.9 vs 86.5% for overall bleedings, *P* = 0.0324; 67.9 vs 86.5% for unscheduled bleedings, *P* <0.001).

Early study withdrawals associated with abnormal bleeding were reported for four (2%) patients during the 13‐cycle study 1 and for three (3%) DRSP 4 mg patients and six (6%) desogestrel patients in the 9‐cycle study 2 (see Table 3).

**Table 3:** Median number of bleeding or spotting days and unscheduled bleeding or spotting days by reference period. Study 1 and study 2 and early study withdrawal associated with abnormal uterine bleeding

	Statistics	Study 1	Study 2	
		DRSP 4 mg (n = 713)	DRSP 4 mg (n = 858)	Desogestrel 0.075 mg (n = 332)
Cycles 2‐4	n/N (%)	11.0 (1.5%)	10.0 (1.2%)^b^	12.0 (3.6%)
Cycles 5‐7	n/N (%)	8.0 (1.1%)	6.0 (.7%)	7.0 (2.1%)
Cycles 8‐10/7‐9^a^	n/N (%)	6.0 (.8%)	6.0 (.7%)	7.0 (2.1%)
Cycles 11‐13	n/N (%)	5.0 (.7%)		
Unscheduled				
Cycles 2‐4	n/N (%)	6.0 (.8%)	5.0 (0.6%)^c^	12.0 (3.6%)
Cycles 5‐7	n/N (%)	5.0 (0.7%)	4.0 (0.5%)^b^	7.0 (2.1%)
Cycles 8‐10/7‐9^a^	n/N (%)	3.0 (0.4%)	4.0 (0.5%)^b^	7.0 (2.1%)
Cycles 11‐13	n/N (%)	3.0 (0.4%)		
Early study withdrawal associated with abnormal bleeding	n/N (%)	30 (4.2%)	28 (3.3%)	22 (6.6%)

Abbreviation: DRSP, drospirenone.

a 8‐10 for Study 1, 7‐9 for Study 2.

b *P* <0.05 for DRSP 4 mg vs desogestrel.

c *P* <0.001 for DRSP 4 mg vs desogestrel.

### 3.5 Safety

No case of deep vein thrombosis or pulmonary embolism was reported. One patient developed an elevated potassium level of 5.7 mmol/L (reference range: 3.5‐5.3 mmol/L) after completion of the study treatment (day 256). The patient did not present any clinical signs of hyperkalemia, an electrocardiogram did not reveal pathological signs, and the event resolved without additional treatment within 1 week. This woman did not differ from the general population regarding clinical baseline features.

The most frequent individual treatment adverse events (TEAEs) were acne (47 cases) and headache (32 cases) in study 1 and abnormal uterine bleeding (38 cases) and acne (26 cases) in study 2. The number of subjects who prematurely terminated the trial was 27.8% in study 1 and 19.8% in study 2. The most frequently reported reasons for early study withdrawal were adverse events (12.3% in study 1 and 9.6% in study 2).

## 4 DISCUSSION

According to the European Medicines Agency guidelines, the number of cycles collected should be at least large enough to give an overall PI with a two‐sided 95%CI such that the difference between the upper limit of the CI and the point estimate is not >1.^15^ For an assumed PI <1.0 the number of cycles needed to fulfil this precision requirement with 90% power was 12 337. The number of evaluable cycles from both trials was 14 329. The PI (95%CI) after correction for additional contraception and sexual activity status as well as method failure PI was also <1: 0.7898 (0.3410 to 1.5562) and 0.9682 (0.4180‐1.9077), respectively. These respective PIs (95%CI) calculated for women aged ≤35 years were slightly higher: 1.0223 (0.4414‐2.0144) and 1.0785 (0.4656‐2.1251).

It is well established that estrogens in combined hormonal contraceptives are the primary cause of the elevated risk of thromboembolic events.^16^ Epidemiological studies have also shown that the progestogen component, when used in combination with estrogens, may be involved in the etiology of venous and arterial diseases.^17^, ^18^ This reflects the influence of progestogens on synthesis, release and activation of pro‐ and anticoagulatory and fibrinolytic factors as well as on the function of platelets, endothelium and possibly smooth muscle cells.^2^

The use of combined contraceptives results in an acceleration of coagulation and fibrinolysis, as demonstrated by Kuhl, Winkler and Schindler, by increasing various markers of hemostasis and fibrin turnover.^2^, ^19^, ^20^ This is induced by the action of ethinylestradiol on hepatic and vascular function and is documented by the rise of sex hormone‐binding globule (SHBG).^21^ Progestogens with pronounced androgenic properties, eg levonorgestrel, may counteract estrogen‐induced changes in the hepatic synthesis of hematological factors; other progestogens with antiandrogenic properties or with neutral androgenetic properties may not.^21^

Drospirenone exhibits a different pharmacokinetic profile when administered together with ethinylestradiol. Although the new formulation with 4 mg DRSP contains 33% more active ingredients than a reference combined oral contraceptive (3 mg DRSP + 0.02 mg ethinylestradiol), the extent of systemic exposure at steady‐state is about 32% less with the new formulation, without dose correction (area under the concentration/time curve within one dosing interval of 24 hours after the last dose in each study period calculated according to the linear trapezoid rule [AUC_0‐24h_, ss] = 77.81%, 90% CI 74.64‐81.12%).^12^ Combined with a reduced *C*_max_, this pharmacokinetic profile of the new formulation may be relevant for similar efficacy and enhanced safety, both characteristics explaining the high efficacy and safety profile found in these clinical trials.

A weakness of the study is the clinical extrapolation on VTE events, as 3333 women‐years cases would have been needed to assess the statistical number of events without any hormonal influence and the total observational period of the studies enrolled 1102 women‐years, as the primary endpoint was the definition of Pearl index.

Eight pregnancies occurred during the treatment and all of them were assessed as due to method failure. The discontinuation rate of 27.8% in study 1 and 19.8% in study 2 was lower or comparable to a clinical trial with other COCs where prematurely trial termination was up to 45%.^22^ The overall PI (95%CI) was 0.7258 (0.3133 to 1.4301), calculated for all women and 0.9332 (0.4029‐1.8387) for women aged ≤35 years (11 145 cycles).

## 5 CONCLUSION

This new DRSP‐only pill provides clinical contraceptive efficacy similar to currently marketed COCs containing DRSP, with a good safety profile, widening the group of women able to use this contraceptive method.^23^

The strengths of these trials were the large program, methodically well conducted, providing clinically meaningful information on a novel POP. The weakness is the number of cycles to assess the PI properly are still not enough, even if indicative, to draw conclusions regarding the risk of VTE.

Future research must focus on the general and widespread use of this new contraceptive method and epidemiological data must be obtained to align the promising results of these two primary studies.

## CONFLICT OF INTEREST

Santiago Palacios is a consultant for Pfizer, Amgen, MSD, Gynea, Procare Health, Bayer, Sérélys Shinogi, Exeltis, Abbott and Gedeon Richter. Enrico Colli and Pedro Antonio Regidor are employees of Exeltis.
